# Hand Hygiene Practices among Medical Students

**DOI:** 10.1155/2012/679129

**Published:** 2012-09-16

**Authors:** Azzam al Kadi, Sajad Ahmad Salati

**Affiliations:** Department of Surgery, College of Medicine, Qassim University, P.O. Box 6655, Buraidah 51452, Saudi Arabia

## Abstract

*Background*. Hand hygiene is a cost-effective method in preventing infection transmission. Hand hygiene practices have been found to be faulty in most healthcare settings. We conducted a study to evaluate the awareness, and compliance of hand hygiene among undergraduate medical students during their clinical phase in Qassim College of Medicine, Saudi Arabia. *Methods*. A questionnaire based on World Health Organization's concept of “Five Moments for Hand Hygiene” was used to evaluate the awareness of the indications for hand hygiene and compliance was observed during Objective Structured Clinical Examination (OSCE) sessions. Sixty students including thirty-six males (60%) and twenty-four females (40%) participated voluntarily in the study. *Results*. The average awareness regarding the positive indications of hand hygiene was 56%. Rest of the 44% of students were either not sure or unaware of the indications of hygiene. Only 29% of students were able to identify all the five indications for hand hygiene in the questionnaire. Compliance as assessed during OSCE sessions was only 17% with no significant difference between the genders. *Conclusion*. It was concluded that serious efforts are needed to improve the hand hygiene practices among medical students.

## 1. Introduction

Hand hygiene is an important healthcare issue globally and is a single most cost-effective and practical measure to reduce the incidence of healthcare-associated infection and the spread of antimicrobial resistance across all settings—from advanced health care systems to primary healthcare centers [1, 2].These infections are the most common adverse events resulting from a stay in the hospital affecting approximately 5 to 10% of hospitalized patients in the developed world, and the burden is larger in underdeveloped nations. In spite of being a very simple action, compliance with hand hygiene among health care providers is as low as less than 40% [3–5]. To address this problem of lack of compliance with hand hygiene, continuous efforts are being made to identify effective and sustainable strategies. One of such efforts is the introduction of an evidence-based concept of “My five moments for hand hygiene” by World Health Organization. These five moments that call for the use of hand hygiene include the moment before touching a patient, before performing aseptic and clean procedures, after being at risk of exposure to body fluids, after touching a patient, and after touching patient surroundings. This concept has been aptly used to improve understanding, training, monitoring, and reporting hand hygiene among healthcare workers [6] in spite of the fact that some recent research has recommended more cautious approach in the universal adoption of this concept [7]. Another strategy is to ensure proper education of the trainee health work force, and in this regard, multiple studies have been conducted to study the hand hygiene practices of nursing and medical students. Such studies are important as the students in their clinical training phase throng the healthcare facilities and can potentially transmit infections besides being the healthcare providers of future when their pattern of training will reflect on their infection control practices. Snow et al. [8] found the medical students to have a low overall rate of hand hygiene. Van De Mortel et al. [9] found the nursing students' hand hygiene knowledge and self-reported practices to be significantly better than that of medical students. Studies have been conducted in Saudi Arabia to study hand hygiene practices in certified healthcare providers [10, 11], but only one significant study has been undertaken where medical students were also evaluated [12]. But in none of these studies WHO's concept of “My five moments for hand hygiene” has been utilized to evaluate hand hygiene practices. Hence the WHO's concept was made the basis of the present study to evaluate hand hygiene awareness and compliance among undergraduate medical students of the Qassim College of Medicine, Saudi Arabia. This study is the first of its kind in the kingdom and is expected to inspire further projects in other medical institutions and in the long run promote the concept of proper hand hygiene among trainee medical students.

## 2. Material and Methods

A cross-sectional study was undertaken from January to March 2012 in the Department of Surgery of College of Medicine, Qassim University, Saudi Arabia, after being approved by the research and ethics committee. On the basis of WHO's concept of “Five Moments for Hand Hygiene” [6], activities commonly undertaken by medical students during clinical phase (4th year of MBBS course) were selected, and a questionnaire was designed (Table 1). 

The compliance of students was assessed during OSCE (Objective Structured Clinical Examination) sessions by the surgeons who were neither involved in the survey nor were in any way related to formative/summative assessment of the students. This strategy was adopted to avoid the possibility of any bias in actual compliance assessment. In these OSCE sessions, alcohol hand rub was displayed beside the simulated patients. Four scenarios based on Moment 1 and Moment 4 were used to assess compliance regarding hand hygiene, and a simple form was used for documentation as shown in Table 2. 

The purpose of the study was explained as per the ethical guidelines of Helsinki, and students were requested to fill the questionnaires after assuring them of the fact that the results had no impact on their final grades in MBBS examinations. The participation of students was voluntary, and the questionnaires were kept anonymous. However, the identifying data of students who opted not to respond to questionnaire was recorded to exclude them from assessment of compliance. The total number of responses was collected, and data was processed and analyzed using SPSS (statistical package for social sciences, version10) and Microsoft Excel-97 software.

## 3. Results

**Figure 1:**
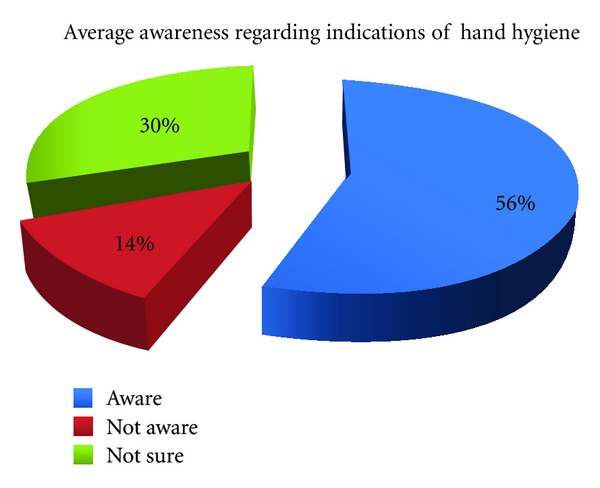
Average hand hygiene awareness among medical students.

A total of eighty-six students were invited to fill the questionnaire. Sixty students (69.76%) agreed to participate and subsequently were enrolled in the study. The participating students included thirty-six males and twenty-four females. The average awareness regarding the positive indications of hand hygiene was 51.7% for male students and 62.5% for female students. Only 29% of students were able to identify all the five indications. There was no significant difference (*P* > 0.05) between two genders. The detailed results are depicted in Table 3 and Figure 1.

**Figure 2:**
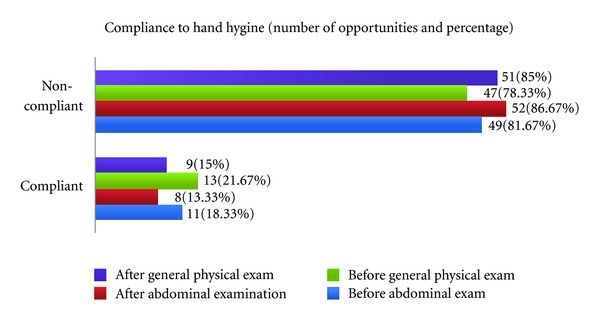
Compliance to hand hygiene among medical students.

Thirty-six male students and twenty-four female students got four potential opportunities each to perform hand hygiene during OSCE sessions. Hand hygiene was performed by male students on 24 out of 144 occasions (compliance—16.7%) and by females on 17 out of 96 occasions (compliance—17.7%) resulting in average compliance of 17% for all students with no statistical difference among genders (*P* < 0.05). The compliance profile to hand hygiene is shown in Figure 2.

## 4. Discussion

Healthcare-associated infection is a very important health issue globally, and hand hygiene is an effective method of infection control. The methods of hand hygiene are widely publicized and simple [1]. Recent studies have found low awareness level regarding hand hygiene among medical students and certified healthcare providers [10–14]. However, the hand hygiene practices have not been studied thoroughly among medical students (trainees) in the Middle East although a few studies have been undertaken on healthcare providers. The present study was aimed to fill this gap and assess the undergraduate medical students' awareness and compliance for hand hygiene. The group studied was in their fourth clinical year that frequently performs activities which must require proper hand hygiene in order not to jeopardize patient's health. 

In our study, 56% of positive indications for hand hygiene outlined in the questionnaire were correctly identified, and 44% students were either unaware or not sure about these moments. This is comparable to the results that have been reported in literature. Van de Mortel et al. [15] observed that 63% of medical students were aware of the correct indications for hand hygiene while Mann and Wood [16] reported awareness in only 56% of students. In our series, only 29% of medical students were able to identify all the indications of hand hygiene. This result is similar to the series of Graf et al. [17], where 33% of students could do so. Graf et al. used seven scenarios where hand hygiene was indicated in five and not indicated in two, but as the present study was pilot project on the subject, we designed a simpler questionnaire based on five common positive indications for hand hygiene. 

 The compliance with hand hygiene among our students was only 17% (*P* < 0.005) as compared to the level of awareness (56%) among the same group of students. These figures are alarmingly low, but on review of literature, we get a similar dismal picture from other studies. Feather et al. [14] studied the hand hygiene practices of 187 candidates during final MBBS OSCE (Objective Structured Clinical Examination) at The Royal London Hospital School of Medicine and Dentistry in UK, and found that only 8.5% of candidates washed their hands after patient contact, although the figure rose to 18.3% when hand hygiene signs were displayed. The situation in healthcare centers of developing countries is even more unacceptable [18]. In an earlier study from Saudi Arabia [12], adherence to hand hygiene was seen in 70% of medical students, 18.8% of nurses, and 9.1% of senior medical staff, but the technique was suboptimal in all. The reasons given by authors for these apparently paradoxical results include limited patient care responsibilities and better undergraduate education and motivation of students on infection control issues and decreasing awareness or conviction with increasing schedules and patients responsibilities as healthcare workers attain seniority. Failure to translate awareness of hand hygiene into compliance has been shown to be a major problem among certified healthcare providers in many other studies also [19].

 Lack of hand hygiene awareness and compliance in medical students has been attributed to many factors. These factors include the following.

### 4.1. Mentors or Role Models

The behavior of students is strongly influenced and molded by their mentor's attitude at the bed side. This has been validated in reference to hand hygiene practices in multiple studies [20, 21]. The role models change with each passing year of training from teachers to senior colleagues and if any of these role models are performing faulty hand hygiene, as is very common in hospital settings, then the students are likely to be less compliant. 

### 4.2. Importance of Hand Hygiene in the Undergraduate Syllabus

 Students are bound to develop faulty hand hygiene practice if the curriculum was not enforced with hand hygiene concepts and skills. One such series is reported by Anwar et al. [18] from a leading medical training center in Pakistan where only 17% of interns and postgraduate medical students were aware of WHO recommendations on hand hygiene and only 4.7% reported to observe correct hygiene before having direct contact with the patients. It is for this valid reason that the Hygiene Liaison Group, UK [21] strongly advocates teaching elementary hygiene practices at medical schools. Even in developed countries, hand hygiene may not get adequate coverage as has been shown by Mann and Wood [16] who examined the infection control knowledge of third year medical students at the University of Birmingham Medical School in UK using a semistructured questionnaire which included a hand hygiene component. The mean hand hygiene knowledge score was 52.3%, while 58% did not know the correct indications for the use of alcohol hand rub.

### 4.3. Beliefs and Attitudes of Students

The Hand Hygiene Liaison Group and the Department of Health in UK have issued guidance stating that hand hygiene compliance reflects the attitudes, behaviors, and beliefs of healthcare personnel. Many authors have addressed this issue [22, 23] in literature stressing the importance of correct hygiene behavior development at the early years of medical education. At this course, students are made to understand effectively the outcomes of proper and improper hygiene. Although faulty behavior and practice by certified health care providers must urgently be corrected, the attitude is not simply rectified. Van de Mortel et al. in 2010 [22] compared the hand hygiene knowledge, beliefs, and practices between nursing and medical students. They found that the nursing students hand hygiene knowledge was significantly higher than that of medical students (*P* < 0.01). Furthermore nursing students had more positive beliefs about hand hygiene (*P* = 0.005). Graf et al. [17] studied the attitudes and beliefs of medical students when they were being promoted from the basic to the clinical phase and noticed a major lack of information regarding the indications of proper hand hygiene. The medical students also believed that the hand hygiene compliance would be even worse at the level of experienced physicians and senior consultants though the seniors are often considered to be role models for trainees.

### 4.4. Use of Gloves

Use of examination gloves in the absence of proper education and guidelines has been found to be a barrier to effective hand hygiene [24–26]. In our study, we found that medical students (36% males and 41.5% females) were unsure about hand hygiene in scenarios (Moments 2 & 3 in Table 3) where they were supposed to wear gloves.

 Multiple methods have been suggested to improve awareness and compliance among students as positive changes in their hand hygiene behavior will translate in improved compliance when they join the health care professionals force and act as mentors for future students [27]. Mathur [1] has proposed the inclusion of regular theoretical education and practical demonstrations on hand hygiene from early on in the curriculum to prime the medical students to these basic health precautions before they take on clinical posts. Mittal et al. [28] have recently proposed a novel method of training hand hygiene. They used germ simulation to illustrate the transmission of bacteria and reported heightened awareness of the importance of hand hygiene and aseptic precautions among students after these training sessions. Fisher et al. [29] suggest formal teaching of hand hygiene in medical schools using a hand-on approach. Feather et al. [14] stress the need for hand hygiene to be made an educational priority and suggest that compliance be made part of assessment tools like OSCE checklists as student learning is highly focused by formative and summative assessments. Scheithauer et al. [30] conducted an observational study in Germany to evaluate the influence of teaching and monitoring on hand hygiene compliance and proposed implementation of regular education and practical training on hand hygiene from early on in the medical studies curricula.

 After this study, we have added and included most of the recommendations from the literature in our teaching methodology including hands on training, incorporation of compliance in OSCE checklists, and increased emphasis on teaching staff incorporating hand hygiene in routine teaching. In addition we propose the incorporation of hand hygiene kits (like alcohol-based hand rubs, hand washes) in skills labs where preclinical medical students get trained on models; the students should be made to observe hand hygiene while handling models so that the importance of this ritual gets imprinted in their developing behaviors. A follow-up study is planned after two years to see the outcome of our changed strategies with respect to teaching of hand hygiene.

## 5. Conclusion

Hand hygiene is the most effective method of preventing transmission of infections. The hand hygiene awareness and compliance among the medical students were found to be very low. Multifaceted and dedicated efforts must be undertaken to rectify this attitude and behavior from early on. Medical schools are highly encouraged to modify and enhance their curriculum in order to improve hand hygiene practices among the students. The improved understanding of infection control and hand hygiene among students is expected to play a major role in curbing disease transmission when the students pass out and join the healthcare work force in future.

## Figures and Tables

**Table 1:** Self-designed questionnaire used for assessment of hand hygiene awareness.

Is hand hygiene (washing with antiseptic soap/alcohol hand rub) recommended?	Yes	No	Not sure
(1) Before abdominal examination.			
(2) Before blood sample extraction with gloved hands.			
(3) After dressing the wound with gloved hands.			
(4) After shaking hands with in-patients at the end of detailed history taking session.			
(5) After touching apparently clean linen bedding or food package in the patient's room.			

**Table 2:** Scenarios used for assessment of hand hygiene compliance.

Opportunities of hand hygiene	Hand-hygiene observed	Hand-hygiene not observed
(1) Before abdominal examination		
(2) After abdominal examination		
(3) Before general physical examination		
(4) After general physical examination		

**Table 3:** Hand hygiene awareness among medical students.

Moments of hand hygiene	Male students (*n* = 36)			Female students (*n* = 24)		
	Yes	No	Not sure	Yes	No	Not sure
(1) Before abdominal examination	28 (77.8%)	2 (5.6%)	6 (16.6%)	18 (75%)	3 (12.5%)	3 (12.5%)
(2) Before blood sample extraction with gloved hands.	15 (41.7%)	7 (19.4%)	14 (38.9%)	12 (50%)	1 (4.2%)	11 (45.8%)
(3) After dressing the wound with gloved hands.	21 (58.3%)	3 (8.3%)	12 (33.3%)	13 (54.2%)	2 (8.3%)	9 (37.5%)
(4) After shaking hands with patients at the end of detailed history taking session	13 (36.1%)	4 (11.1%)	19 (52.8%)	15 (62.5%)	3 (12.5%)	6 (25%)
(5) After touching apparently clean linen bedding or food package in the patient's room.	16 (44.4%)	14 (38.9%)	6 (16.7%)	17 (70.8%)	2 (8.3%)	5 (20.8%)

Average	18.6 (51.7%)	6 (16.7%)	11.4 (31.7%)	15 (62.5%)	2.2 (9.2%)	6.8 (28.3%)
